# Plasmacytoid dendritic cells appear inactive during sub-microscopic *Plasmodium falciparum* blood-stage infection, yet retain their ability to respond to TLR stimulation

**DOI:** 10.1038/s41598-017-02096-2

**Published:** 2017-06-01

**Authors:** Jessica R. Loughland, Gabriela Minigo, Derek S. Sarovich, Matt Field, Peta E. Tipping, Marcela Montes de Oca, Kim A. Piera, Fiona H. Amante, Bridget E. Barber, Matthew J. Grigg, Timothy William, Michael F. Good, Denise L. Doolan, Christian R. Engwerda, Nicholas M. Anstey, James S. McCarthy, Tonia Woodberry

**Affiliations:** 1 Menzies School of Health Research, Darwin, Australia and Charles Darwin University, Darwin, Australia; 2 Centre for Animal Health Innovation, Faculty of Science, Health, Education and Engineering, University of the Sunshine Coast, Sippy Downs, Queensland Australia; 3 Australian Institute of Tropical Health and Medicine, James Cook University, Cairns, Australia; 4 QIMR Berghofer Medical Research Institute, Brisbane, Australia; 5 Infectious Diseases Unit, Queen Elizabeth Hospital, Kota Kinabalu, Sabah Malaysia; 6 Sabah Department of Health, Kota Kinabalu, Sabah Malaysia; 7 Griffith University, Gold Coast, Australia; 8 Royal Darwin Hospital, Darwin, Australia; 9 Royal Darwin Hospital, Darwin, Australia

## Abstract

Plasmacytoid dendritic cells (pDC) are activators of innate and adaptive immune responses that express HLA-DR, toll-like receptor (TLR) 7, TLR9 and produce type I interferons. The role of human pDC in malaria remains poorly characterised. pDC activation and cytokine production were assessed in 59 malaria-naive volunteers during experimental infection with 150 or 1,800 *P. falciparum-*parasitized red blood cells. Using RNA sequencing, longitudinal changes in pDC gene expression were examined in five adults before and at peak-infection. pDC responsiveness to TLR7 and TLR9 stimulation was assessed *in-vitro*. Circulating pDC remained transcriptionally stable with gene expression altered for 8 genes (FDR < 0.07). There was no upregulation of co-stimulatory molecules CD86, CD80, CD40, and reduced surface expression of HLA-DR and CD123 (IL-3R-α). pDC loss from the circulation was associated with active caspase-3, suggesting pDC apoptosis during primary infection. pDC remained responsive to TLR stimulation, producing IFN-α and upregulating HLA-DR, CD86, CD123 at peak-infection. In clinical malaria, pDC retained HLA-DR but reduced CD123 expression compared to convalescence. These data demonstrate pDC retain function during a first blood-stage *P. falciparum* exposure despite sub-microscopic parasitaemia downregulating HLA-DR. The lack of evident pDC activation in both early infection and malaria suggests little response of circulating pDC to infection.

## Introduction

Innate recognition of *Plasmodium* parasites is critical for the induction of appropriate anti-malarial immune responses^1^. Plasmacytoid dendritic cells (pDC) are important mediators of both innate and adaptive immune responses (reviewed in ref. 2). Human pDC are HLA-DR^+^ blood cells that lack the lineage-associated markers CD3, CD19, CD14, CD56, CD11c and express high levels of the IL-3 receptor alpha chain CD123. Unlike myeloid DC, pDC express toll-like receptor (TLR) 7 and TLR9 thereby allowing recognition of single-stranded RNA^3^ and DNA^4, 5^, respectively. Upon recognition of pathogen nucleic acids, pDC produce large amounts of immune regulatory type I interferons (IFNs). pDC MHC class I and II molecules allow pDC antigen-presentation to CD8^+^ T cells (cross-presentation)^6^ and CD4^+^ T cells^7^. In addition, pDC regulate the generation of plasma cells and antibody responses^8, 9^.

The role of pDC in *Plasmodium* infection remains unclear. *Plasmodium* parasite protein-DNA complexes are thought to activate TLR9^10, 11^, and *Plasmodium*-derived RNA can trigger type I IFN responses in a TLR7-dependent^12^ and -independent fashion^13^ in mice. The *Plasmodium* agonist of human TLR7 remains to be identified.

Murine models of malaria show differing pDC responses, depending on the mouse strain and parasite species. Across mouse strains, pDC upregulate CD86^12, 14, 15^, MHC class II^14^ or CD40^12^ following *Plasmodium* infection. Production of type I IFN by pDC has been demonstrated at the transcriptional^14, 16^ and protein level^15, 17^ following infection with three rodent-infecting *Plasmodium* species. The relevance of pDC activation in murine malaria is debated. In the *P. chabaudi* model of chronic infection and the *P. berghei* model of cerebral malaria, pDC are considered not relevant for the control of blood parasitaemia or disease outcome despite their activation^12, 14, 16^. Most recently, pDC derived type I IFN early during infection has been reported as essential for the induction of protective immune responses against lethal *P. yoelii* YM^17^, and *Plasmodium* parasites may prevent early pDC type-I IFN release by activating cross-regulatory type-I IFN pathways^17^.

Much less is known about the role of pDC in human malaria, mainly due to limitations intrinsic to human studies and restrictions to peripheral blood. There is a consensus that blood pDC numbers decline in acute clinical malaria^18–22^. However, it is unclear whether pDC loss is due to migration^18^ or cell death^19^. Evaluation of pDC maturation in malaria has been limited to the assessment of surface CD86 and HLA-DR. CD86 upregulation has been reported in children with severe malaria^15^, *P. falciparum* infected pregnant women^20^ and some patients with acute vivax malaria^23^, whilst HLA-DR is not upregulated in severe malaria^15, 24^. In agreement with this, a Malian study of two ethnic groups with differing susceptibility to symptomatic malaria shows in *P. falciparum* infected malaria-susceptible Dogon, pDC downregulate HLA-DR expression, lack upregulation of CD86 and show impaired responsiveness to TLR9 stimulation. Circulating pDC in *P. falciparum* infected Fulani, an ethnic group less susceptible to symptomatic malaria, on the contrary, display a mature phenotype with upregulated CD86 and HLA-DR expression and strong TLR9 responsiveness^25^. These studies, together with recent murine studies^15, 17^, suggest that pDC may play an integral part in the acquisition of clinical immunity.

Surprisingly, our recent report on early *P. falciparum* blood-stage infection found pDC to be minor contributors to the early IFN-α response^26^. We have also shown, circulating pDC decline early during the pre-patent phase of a primary infection^27^. These findings, together with the recent report of inhibited type I IFN release from pDC during early *P. yoelii* infection^17^ and our past identification of profoundly impaired CD1c^+^ myeloid DC in very early *P. falciparum* infection^28^, prompted us to investigate pDC activation and function in more detail. Controlled human malaria infection (CHMI), provides the unique ability to assess host responses during *P. falciparum* blood-stage infection with each individual’s baseline response before infection. To characterise pDC activation and function in a primary *P. falciparum* blood-stage infection, we isolated highly purified pDC for gene expression analysis, monitored the expression of co-stimulatory markers CD86, CD80 and CD40 and measured pDC responsiveness to TLR7 and TLR9 stimulation at baseline and at peak-infection.

## Results

### Circulating pDC gene expression is predominantly stable after primary pre-patent *P. falciparum* CHMI

We isolated blood pDC at >98% purity from five volunteers at baseline and at peak parasitaemia following intravenous infection with 1,800 pRBC (n = 5, median parasitaemia 4,754/mL [1,320–11,929]). Transcriptional gene expression changes were investigated by RNA sequencing as described in the methods. Globally, there were only modest changes (false detection rate, FDR < 0.07) in pDC gene expression. Eight gene transcripts had significantly decreased or increased gene expression (FDR < 0.07, Supplementary Figure 1), when baseline was compared to peak-infection. A summary of the top 10 genes that increased and top ten genes that decreased expression at peak-infection is presented in Table 1. No *P. falciparum* RNA signal was detected (data not shown).

**Table 1:** pDC gene expression summary.

	**FDR**	**P-value**
**Genes Upregulated**		
**NLRC5**	**0.02**	**0.000005**
**C14orf119**	**0.03**	**0.00001**
**TSG101**	**0.04**	**0.00003**
GPATCH11	0.10	0.0001
NMI	0.19	0.0004
HLA-F	0.19	0.0004
ENP22	0.19	0.0004
KCNA5	0.25	0.0006
ENSG021574	0.28	0.0008
SAC3D1	0.37	0.002
**Genes Downregulated**		
**DMBT1**	**1.05E** ^**−15**^	**9.8 × 10** ^**20**^
**AREGB**	**0.03**	**0.00001**
**RNF139**	**0.02**	**0.000005**
**CRYM**	**0.03**	**0.00001**
**BAG3**	**0.067**	**0.00006**
ENSG0253333	0.09	0.00008
ENSG0257513	0.19	0.0004
CXCR4	0.11	0.0002
AREG	0.23	0.0005
KRTCAP3	0.33	0.001

Genes in bold have a False Detection Rate (FDR) < 0.07.

### Circulating pDC of immature phenotype during early *P. falciparum* CHMI

**Figure 1:**
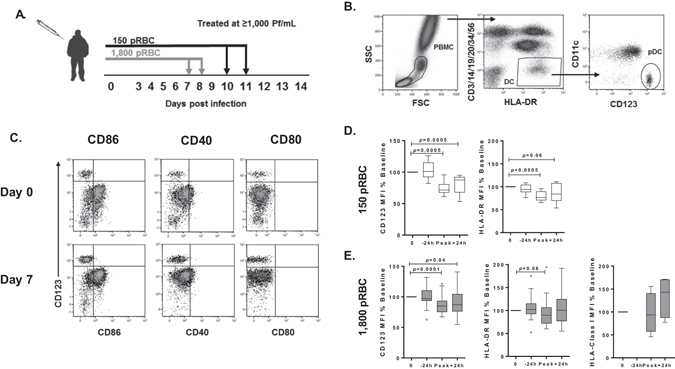
Circulating pDC are not activated in *P. falciparum* CHMI. (**A**) Schematic of clinical trial cohorts 150 pRBC (black) and 1,800 pRBC (grey), arrows indicate antimalarial treatment. (**B**) Fresh whole blood gating strategy for pDC. pDC were identified as negative for lineage markers (CD3, CD14, CD19, CD20, CD34 and CD56), HLA-DR^+^ (2^nd^ panel), CD11c^−^ and CD123^+^ (3^rd^ panel). (**C**) Representative flow cytometry plots from one participant showing CD86, CD40 and CD80 pDC expression before infection (day 0) and at peak-infection. Gated on total DC as shown in Fig. 1B 2^nd^ panel. (**D**) Longitudinal pDC CD123 and HLA-DR expression after 150 pRBC infection. (**E**) Longitudinal pDC CD123, HLA-DR and HLA-A,B,C expression after 1,800 pRBC infection. Box plots show the minimum, maximum, median and interquartile range for data from all participants (turkey plot). Abbreviations FSC, forward scatter, SSC, side scatter, MFI, median fluorescence intensity.

**Figure 2:**
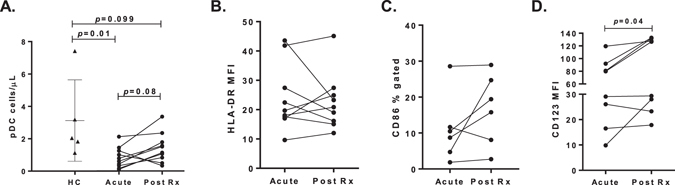
Characterisation of circulating pDC in clinical patients infected with *P. falciparum*. (**A**) The absolute number of circulating pDC in 5 healthy controls (black triangles) and 10 patients with acute *P. falciparum* and at convalescence, 14 to 28 days after antimalarial drug treatment (Post Rx). (**B**) Longitudinal HLA-DR expression (MFI) on pDC of patients with acute *P. falciparum* malaria. (**C**) Longitudinal CD86 percent positive pDC of patients with acute *P. falciparum* malaria. (**D**) Longitudinal CD123 expression (MFI) on pDC of patients with acute *P. falciparum* malaria. Mann-Whitney t-test was used for comparison between healthy controls and clinical malaria patients. Wilcoxon matched-paired test was used for comparison between acute malaria and convalescence (Post Rx). Abbreviations: MFI, median fluorescence intensity.

To phenotypically characterise pDC during early *P. falciparum* infection we assessed maturation markers; CD86, CD40, CD80, HLA-A,B,C, HLA-DR and CD123 (IL-3Rα) expression on pDC before and at peak-infection for participants infected with either 150 pRBC or 1,800 pRBC. Mean parasitaemia was determined by PCR^29^. Participants administered 150 pRBCs reached peak parasitaemia on day 10 (n = 1, parasitaemia 12,750/mL), or day 11 (n = 11, median parasitaemia 2,380/mL [IQR 696–5085]), whereas participants administered 1,800 pRBCs reached peak parasitaemia on day 7 (n = 23, median parasitaemia 6,877/mL [IQR 4,759–12,813]) or day 8 (n = 24, median parasitaemia 4,970/mL [IQR 666–18,065]) (Fig. 1A). Circulating pDC were identified in whole blood as lineage marker negative (CD3, CD14, CD19, CD20, CD34 and CD56), HLA-DR^+^, CD11c^−^ and CD123^+^ (Fig. 1B). In fresh whole blood, less than 1% of pDC expressed CD86, CD40 or CD80 before infection or at peak parasitaemia (Fig. 1C). pDC expression of CD123, the alpha subunit to the IL-3 receptor (IL-3Rα)^30^, frequently used to identify pDC subsets^31^, significantly reduced at peak parasitaemia and failed to recover by 24 hours after antimalarial treatment (Fig. 1D,E). HLA-DR expression on pDC also reduced in both cohorts at peak parasitaemia (Fig. 1D,E). Additionally, HLA-A,B,C expression increased on pDC in the 1,800 pRBC cohort, although the increase was not statistically significant (Fig. 1E). In accordance with previous reports^32, 33^, pDC did not uptake FITC-dextran particulate antigen at baseline and this did not change with infection (data not shown). In clinical malaria, circulating pDC showed a similarly immature phenotype lacking upregulation of CD86 or HLA-DR and significantly reduced CD123 expression during acute disease compared to convalescence (Fig. 2).

### Reduced circulating pDC number is associated with increased active caspase-3 expression

**Figure 3:**
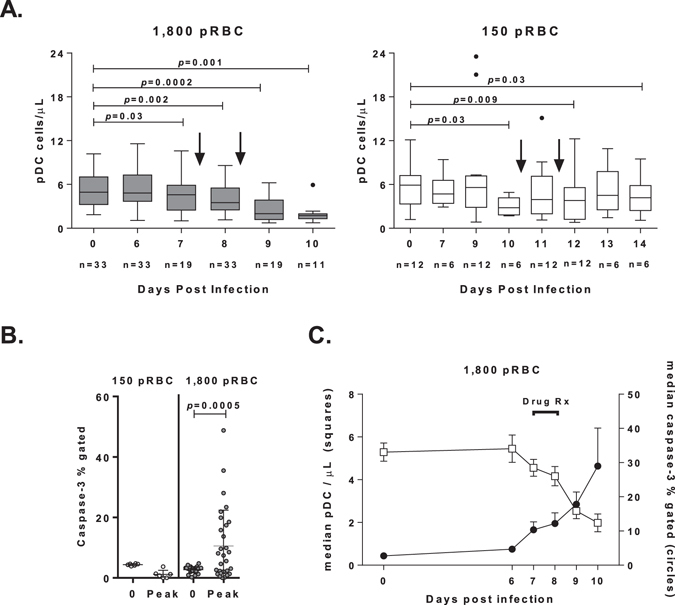
Increased pDC active caspase-3 detection. (**A**) The absolute number of circulating pDC after 1,800 pRBC infection; arrows indicate day of antimalarial treatment (n = 9 (day 7), n = 24 (day 8)). The absolute number of circulating pDC after 150 pRBC infection; arrows indicate day of antimalarial treatment (n = 1 (day 10), n = 11 (day 11)). Box plots show the minimum, maximum, median and interquartile range for data from all participants (turkey plot). Data points outside of box plots represent patients which were outliers. The number of individual data points for each day is given below the x-axis. (**B**) Percentage of pDC positive for active caspase-3 after 150 pRBC (n = 6, open circles) or 1,800 pRBC (n = 30, closed circles) infection. The horizontal line and whiskers represent the median and inter-quartile range, Wilcoxon matched-paired test was used for comparison between day 0 and day 7–8 (peak) or day 10–11 (peak). (**C**) Mean (+/−SEM) number of circulating pDC (white squares) and proportion of active caspase-3 positive pDC (black circles) after 1,800 pRBC infection n = 30. Abbreviations: FSC, forward scatter SSC, side scatter, MFI, median fluorescence intensity, SEM, standard error of the mean.

Circulating pDC numbers were determined longitudinally in both the 1,800 pRBC and 150 pRBC infection cohorts. pDC significantly declined at peak parasitaemia and continued to fall 24 hours after antimalarial drug treatment in the 1,800 pRBC infection cohort (Fig. 3A **, grey bars**). In the lower 150 pRBC cohort, pDC number fell significantly 24 hours pre and post peak parasitaemia (Fig. 3A **, white bars**). After antimalarial drug treatment, pDC numbers remained low at 48 and 72 hours (Fig. 3A). To determine if the loss of pDC was attributable to an increase in pDC apoptosis, circulating pDC were examined for the presence of the active form of caspase-3, a hallmark apoptosis marker. Caspase-3 is an executioner caspase, essential to both intrinsic and extrinsic apoptotic pathways^34^. There was no evidence of active caspase-3 in blood pDC following infection with 150 pRBC (n = 6; median 1% [IQR 1–3%] caspase-3, Fig. 3B). In contrast, following 1,800 pRBC infection, we observed significantly increased active caspase-3 in pDC (n = 30; median 6.5% [IQR 2–18] caspase-3, Fig. 3B **)**. Longitudinal changes in circulating pDC number and active caspase-3 expression were assessed in the 1,800 pRBC cohort, 48 hours before peak parasitaemia (day 6) to 48 hours after antimalarial treatment (day 10). As *P. falciparum* infection progressed, the number of circulating pDC declined, while pDC expressing caspase-3 increased (Fig. 1C). Thus, we show for the first time that pDC are apoptotic during the higher dose *P. falciparum* infection.

### Circulating pDC retain the ability to produce IFN-α and TNF during controlled *P. falciparum* infection

**Figure 4:**
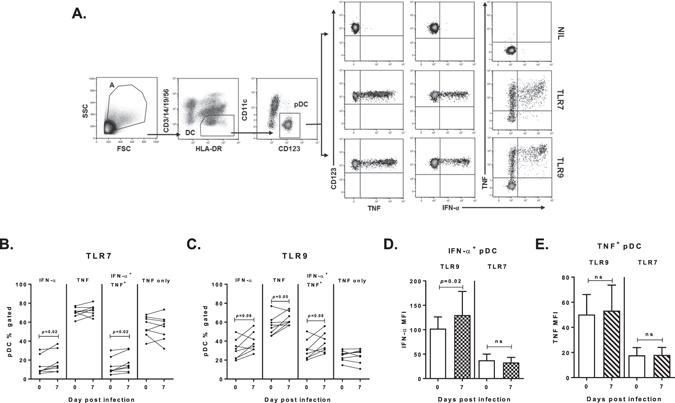
pDC cytokine responses to TLR stimulation. (**A**) Representative staining of circulating pDC in PBMC evaluated for intracellular cytokines. pDC were identified as lineage (CD14, CD3, CD19 and CD56)^−^, HLA-DR^+^, CD11c^−^ and CD123^+^. Intracellular cytokine production by *ex vivo* pDC on day 0 (IFN-α and TNF) in three conditions: no stimulation (top panel), TLR7 stimulation (middle panel) and TLR9 stimulation (bottom panel). (**B**) IFN-α and TNF produced by pDC on day 0 and day 7 after TLR7 stimulation, n = 8. (**C**) IFN-α and TNF produced by pDC on day 0 and day 7 after TLR9 stimulation, n = 8. (**D**) IFN-α MFI of IFN-α producing pDC after TLR7 or TLR9 stimulation, n = 8. (**E**) TNF MFI of TNF producing pDC after TLR7 or TLR9 stimulation, n = 8. Data are represented as median and interquartile range. The Wilcoxon matched-paired test was used for comparison between day 0 and day 7. Abbreviations: FSC, forward scatter SSC, side scatter, MFI, median fluorescence intensity.

To assess pDC function during early *P. falciparum* infection we evaluated IFN**-**α, TNF, IL-10 and IL-12 cytokine production and maturation in response to TLR stimulation (n = 8, Fig. 4A). pDC were identified as lineage^−^, HLA-DR^+^, CD11c^−^ and CD123 (IL-3Rα)^+^ (Fig. 4A). At peak-infection, more pDC produced IFN-α in response to TLR7 (*p* = 0.02, Fig. 4B) or TLR9 stimulation (*p* = 0.08, Fig. 4C), when compared to baseline hence, the ability of pDC to produce IFN-α was not diminished during early *P. falciparum* blood-stage infection. IFN-α producing pDC always co-produced TNF (Fig. 4A) and infection did not change the proportion of pDC producing TNF alone (Fig. 4B,C). A comparison between cytokine MFI at peak parasitaemia and baseline (day 0) showed that TLR9-stimulated pDC expressed more IFN-α at peak-infection (Fig. 4D), whilst TNF production remained unchanged (Fig. 4E). No IL-10 or IL-12 production by pDC was detected. The same pattern of cytokine response (dual IFN-α/TNF and no IL-12 or IL-10) was observed when PBMC or fresh whole blood was used in the assay.

**Figure 5:**
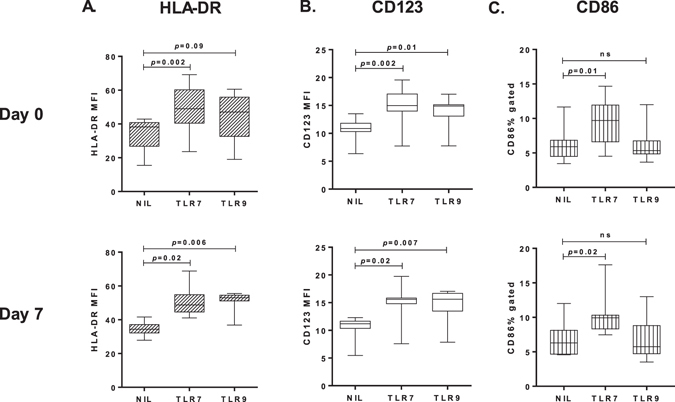
Increased HLA-DR, CD123 and CD86 expression on pDC upon TLR stimulation. (**A**) pDC HLA-DR expression (**B**) pDC CD123 expression and (**C**) pDC CD86 percent gated on day 0 or day 7, in three conditions: no stimulation and after TLR7 or TLR9 stimulation, n = 8. Box plots show the minimum, maximum, median and interquartile range for data. Multiple comparisons test (1-way ANOVA) was used for comparison between no stimulation and TLR stimulation.

Circulating pDC significantly increased HLA-DR and CD123 surface expression upon TLR7 or TLR9 stimulation, at both baseline and peak parasitaemia (n = 8, Fig. 5A,B). CD86 expression increased significantly following TLR7 stimulation at both baseline and peak parasitaemia, but not upon TLR9 stimulation with CpGA (Fig. 5C), as previously reported^35^. There was no significant change in pDC HLA-DR, CD86 or CD123 expression, either without stimulation (NIL) or following TLR7 or TLR9 stimulation, when baseline was compared to peak-infection in eight individuals.

## Discussion

Here, we have shown pDC are not activated and remain functional during early *P. falciparum* infection. To the best of our knowledge, this is the first study to sequence RNA from highly purified pDC and compare gene expression before and at the peak of a primary *P. falciparum* CHMI. Unexpectedly, we found pDC gene expression to be largely stable in early sub-microscope infection. The observed minimal change in gene expression, together with an absence of activation/maturation phenotype by flow cytometry suggests, circulating blood pDC are not activated, yet remain functional during pre-patent blood-stage infection.

While pDC gene expression was largely unchanged during early infection, a panel of eight genes significantly increased or decreased at peak-infection. The greatest increase in gene expression was seen in NLRC5 (NOD-like receptor (NLR) family, caspase recruitment (CARD) domain containing 5), an IFNγ-inducible transcriptional coactivator of MHC class I gene expression also known as CITA (MHC class I transactivator)^36^. NLRC5 is the master regulator of MHC class I gene expression and activates most of the key components of the MHC class I antigen presentation pathway^37^. In line with this, non-classical MHC class I HLA-F was in the top 10 upregulated genes in this study, although with a FDR > 0.07. Increased HLA class I protein expression was detected on the day after treatment, although not statistically significant. Together these findings suggest that pDC upregulate their MHC class I antigen presentation machinery during early *P. falciparum* infection, whether this leads to cross-presentation of parasite antigen to activate CD8^+^ T cells^38^ or makes them targets for elimination by cytotoxic CD8^+^ T cells remains unclear. pDC have been suggested to act as a reservoir for *Plasmodium* in mice^39^; however, we detected no *Plasmodium* RNA in isolated pDC to support this previous finding, suggesting that this does not occur in human pDC or that our methods lacked the sensitivity to identify *Plasmodium* RNA. Although still controversial, NLRC5 has also been reported to modulate type I IFN responses in various human and murine cell types^40^. Future studies into the effects of NLRC5 in malaria are warranted.

Amongst the downregulated genes was BAG3 (BCL2 associated athanogene gene 3), an anti-apoptotic co-chaperone protein^41^. Down-modulation of BAG3 in HIV-infected microglial cells results in accumulation of active caspase-3^42^. Here we show active caspase-3 detection by flow cytometry in pDC increased in the 1,800 pRBC cohort during early *P. falciparum* infection and was associated with pDC loss from the circulation. pDC loss equally occurred in the 150 pRBC cohort yet, this did not coincide with active caspase-3 detection. Whether this apparent lack of apoptosis in the 150 pRBC cohort is due to differing parasitaemia on the day of treatment or to the longer exposure to a very low parasite dose remains unclear. Very low doses of *P. falciparum* blood-stages have previously been shown to induce host immunity^43^. The reduction in blood pDC numbers during *Plasmodium* infection is in accordance with previous reports^18–23, 27^. Additionally, we show for the first time, pDC reduce CD123, IL-3α receptor expression in *P. falciparum* CHMI and in acute falciparum malaria, suggesting this effect is independent of the route of infection. Whether this reduction impacts responsiveness to IL-3, a T cell derived glycoprotein that supports the viability and differentiation of haematopoietic cells^44^ remains to be determined. Studies of isolated pDC from healthy adults report an increase in pDC CCR7 expression after *in vitro* stimulation with high concentration of *P. falciparum* schizont antigen^18^, suggesting pDC increase their ability to migrate to lymphoid organs following parasite contact. Whilst we did not detect any changes in CCR7 gene expression in this early infection, gene expression of CXCR4 was downregulated. pDC constitutively express CXCR4 on their surface which is required for their development^45^ but also allows them to enter lymphoid tissues via high endothelial venules^46^. Downregulation of CXCR4 transcription may indicate less repopulation from the bone marrow, reduced ability to migrate to lymphoid tissue or indeed very early activation, as pDC downregulate CXCR4 gene expression following *in vitro* stimulation with Influenza virus^47^. In the *P. yoelii* model of severe malaria, pDC activation occurs very early during blood-stage infection^15, 17^ and activated type I IFN producing pDC reside predominantly in the bone marrow and blood of *P. yoelii* YM infected mice^15^ and not in the spleen as previously suggested^14, 48^. Conclusive evaluation of dendritic cell migration in human malaria will need to be addressed by researchers with access to *Plasmodium* infected human spleen tissue.

Blood pDC are important for maintaining a balance between immunity and immunopathology in viral infections^2^. Early production of type I IFNs by pDC are needed for containment of virues in systemic infections such as HSV-1^49^. However, pDC derived type I IFNs become less important later on in the infection, as other host cell responses become more dominent (reviewed in ref. 2). Moreover, pDC may contribute to the chronicty of HIV infection through dysregulated activation and type I IFN production^50^. The beneficial or detrimental effects of pDC and type I IFNs in infection may depend on the timing of their action. In lethal *P. yoelii* YM infection, early type I IFN production during infection is essential for the induction of protective immune responses^17^. In contrast, type I IFN production in *P. berghei* ANKA infection, leads to suppression of CD4^+^ T cell adaptive immune responses^51^, although the cellular sources of IFN-α were not identified. Whether pDC activation is desirable or detrimental in human malaria is still not clear.

Assessment of rare human dendritic cell subsets is difficult, and accessing longitudinal human malaria samples is even more challenging. To gain insights into human pDC function in malaria, we took advantage of the CHMI, which allowed longitudinal assessment of pDC function in malaira naive volunteers. Despite loss and apparent apoptosis, pDC retained the ability to respond to TLR stimulation. In contrast, during this same time frame of early pre-patent *P. falciparum* infection peripheral blood CD1c^+^ mDC are dysfunctional and unable to upregulate HLA-DR following TLR stimulation^28^. Together, our studies suggest that DC subsets are differentially affected during *P. falciparum* blood-stage infection and these differences should be considered when evaluating dendritic cells in *Plasmodium* infection and malaria. Evidence of a functional human dendritic cell subset in *P. falciparum* infection is encouraging, as pDC may orchestrate downstream adaptive immune responses during infection.

## Methods

### *P. falciparum* CHMI

Written and informed consent was obtained from 59 volunteers who participated in a series of phase Ib clinical trials testing the efficacy of antimalarial drugs. Blood-stage *P. falciparum* infection was initiated by inoculation of trial parasite doses, 150 (n = 12) or 1,800 (n = 47) parasitised red blood cells (pRBC) as previously described^29^. Anticoagulated blood was collected for immunologic assessment at specific time points, before and during blood-stage infection. Antimalarial drugs were administered when volunteers reached a pre-determined parasitaemia of ≥1,000 parasites/mL (day 10 or 11; 150 pRBC and day 7 or 8; 1,800 pRBC) (Fig. 1A). Fresh whole blood or cryopreserved peripheral blood mononuclear cells (PBMCs) were used in flow cytometric assays. PBMCs were processed within 2 hours of collection. Experimental methods and studies were approved by Human Research Ethics Committees of QIMR Berghofer Medical Research Institute (P1479) and the Human Research Ethics Committees of the NT Department of Health and Menzies School of Health Research (HREC 10/1431). All experimental methods were carried out in accordance with the NHMRC National Statement on Ethical Conduct of Human Research. Written and informed consent was obtained from all participants in the clinical trials. Clinical Trial Registrations; ACTRN12611001203943- registered 23/11/2011; ACTRN12612000323820- registered 21/03/2012; ACTRN12612000814875- 03/08/2012; ACTRN12613000565741-registered 17/05/2013; ACTRN12613001040752- 18/09/2013; NCT02281344- registered 30/10/2014. Details of the clinical trial protocol and clinical trial registrations are reported elsewhere^29, 52^.

### Acute malaria

The number and phenotype of pDC were assessed in cryopreserved PBMCs collected from adults with uncomplicated *P. falciparum* malaria (n = 10) as part of a pathophysiology study at Queen Elizabeth 1 and Kudat District Hospitals in Sabah, Malaysia^53^. PBMC samples were collected prior to commencing treatment, and again 14 to 28 days in a prospective fashion, after antimalarial drug treatment (Table 2). PBMCs from family or friends of patients were evaluated as controls (n = 5). Written informed consent was obtained from participants and the study was approved by the Human Research Ethics Committees of the NT Department of Health and Menzies School of Health Research (HREC 10/1431) and the ethics committee of the Malaysian Ministry of Health (NMRR 10 754 6684). All experimental methods were carried out in accordance with the NHMRC National Statement on Ethical Conduct of Human Research.

**Table 2:** Acute malaria cohort.

	Healthy Control	Acute *P. falciparum*	Post treatment (Rx)
Patients	5	10	10
Age	48 [19–50]	29 [18–45]	
Gender (%M)	40%	60%	
Parasitaemia	.	18,880 [3,077–26,591]	.
WCC (10^3^/mL)	7.7 [5.3–10.3]	5.6 [5.0–7.1]	7.9 [6.9–9.4]
Lymphocyte (10^3^/mL)	2.2 [1.7–2.9]	1.2 [0.8–1.5]*	2.3 [1.0–3.3]
Monocyte (10^3^/mL)	0.4 [0.3–0.7]	0.6 [0.5–1.2]	0.6 [0.4–1.0]

Values show the median and [interquartile range].

Comparison between healthy controls and acute malaria was performed using a Mann-Whitney t-test, *p ≤ 0.05.

### Whole blood pDC and PBMC enumeration and activation

pDC were characterised as lineage (CD3, 14, 19, 20, 34, 56)^−^, HLA-DR^+^, CD11c^−^, CD123^+^ (Fig. 1B). In brief, 200 µL of blood or 3 million PBMC were stained with surface antibodies; CD3 (HIT3a), CD14 (HCD14), CD19 (HIB19), CD20 (2H7), CD34 (561), CD56 (HCD56), HLA-DR (L243), CD11c (B-Ly6), CD123 (6H6), CD86 (2331, FUN-1), CD80 (2D-10), CD40 (5C3) and HLA-A,B,C (W6/32). All antibodies were purchased from BD Biosciences or Australian Biosearch, Biolegend. For whole blood, RBC were lysed with FACS lysing solution (BD, USA) and cells fixed with 1% (w/v) paraformaldehyde in phosphate-buffered saline. Absolute numbers of pDC were determined by adding automated lymphocyte and monocyte counts (10^9^ cells/L), dividing the sum by 100, multiplying the percentage of pDC, and multiplying the product by 1,000 to give the cell count/µL.

### Apoptosis

Intracellular active caspase-3 was measured as previously described^54^. Briefly, 200 µL or 1,000 µL of blood was stained with surface antibodies, RBC were lysed with FACS lysing solution (BD, USA), cells were permeabilised using 1 × BD Perm/Wash™ (BD, USA) and stained with active caspase-3 antibody (C92-605, BD USA).

### FITC-dextran uptake

pDC phagocytosis was assessed by uptake of 1 mg/mL FITC-dextran (Sigma, USA) after 60 min at 37 °C, or on ice as a control, and expressed as delta median fluorescence intensity (ΔMFI) (i.e. [MFI of cells at 37 °C]- [MFI control cells on ice]).

### Intracellular cytokine staining

Cytokine production was assessed in PBMCs (2 million per well in RPMI 10% FCS (Invitrogen, USA)) or 1000 µL of WB stimulated with TLR agonists; TLR7: Imiquimod 2.5 µg/mL and TLR9: CpG ODN2216 50 ug/mL (Sigma-Aldrich, USA). Protein transport inhibitor (Brefeldin A, BD, USA) was added after 2 h at 37 °C, 5% CO_2_. At 6 h, cells were stained to identify pDC (including CD86 (IT2.2)), washed with 2% FCS/PBS, cells permeabilised with 1x Perm/Wash™ and stained with intracellular anti-TNF-α (MAB11), IL-12/IL-23p40 (C11.5), IL-10 (JES3-9D7), and IFN-α (LT27:295).

FACS data were acquired using a FACSCanto™ II (BD, USA) or Gallios™ (Beckman and Coulter, USA). Data were analysed using Kaluza® 1.3 (Beckman Coulter, USA).

### pDC isolation and RNA Sequencing

RNA sequencing was performed on paired samples collected prior to and at peak-infection from five subjects experimentally infected with 1,800 *P. falciparum* pRBC. pDC were isolated from fresh PBMCs using the Human Diamond Plasmacytoid Dendritic Cell Isolation Kit (Miltenyi Biotec, Gladbach, Germany). In brief, pDC were enriched according to the manufacturer’s instructions using CD304 (BDCA-4) conjugated microbeads and washed over MACs isolation columns. Purity of isolated pDC cell populations was checked using flow cytometry (day 0; median 99.3% [IQR 99.1–99.7%], day 7–8 median 99.6% [IQR 99.3–99.7%]) and the number of isolated pDC was estimated (day 0; median 14,336 cells [IQR 7,487–31,590 cells], day 7–8 median 38,491 cells [IQR 8,419–66,622 cells]. Isolated pDC were resuspended in RNAprotect (Qiagen, Australia) and stored immediately at −80 °C. RNA extraction and RNA-sequencing of five paired participant samples were conducted by Macrogen^©^ (Seoul, Korea) using the Illumina TruSeq stranded mRNA LT sample kit and the HiSeq 2500 instrument. Transcriptome data were analysed using a modified version of an existing variant detection pipeline^55^ consisting of software STAR aligner^56^, samtools^57^, HTSeq^58^, and DESeq2^59^. Reads were first aligned to human reference genome GRCh37 using STAR with the gene model set to gencode v19 annotation and quantmode set to TranscriptomeSAM. The alignment files were sorted with samtools and the resultant reads input to HTSeq to generate raw read counts using the union overlap resolution mode. Read counts were input to DESeq2 and a paired analysis performed for the five participants with baseline and peak parasitaemia data.

### Statistics

Statistical analyses were undertaken using GraphPad Prism 6 (Graphpad Software Inc., USA). The Wilcoxon matched-pairs sign rank test was used to compare longitudinal data and a multiple comparisons test (1-way ANOVA) was used for comparison between no stimulation and TLR stimulation.

## Electronic supplementary material

Supplementary Information

## References

[1] Eriksson E, Sampaio N, Schofield L (2013). Toll-Like Receptors and Malaria–Sensing and Susceptibility. J Trop Dis.

[2] Swiecki M, Colonna M (2015). The multifaceted biology of plasmacytoid dendritic cells. Nature Reviews Immunology.

[3] Diebold SS, Kaisho T, Hemmi H, Akira S, e Sousa CR (2004). Innate antiviral responses by means of TLR7-mediated recognition of single-stranded RNA. Science.

[4] Bauer S (2001). Human TLR9 confers responsiveness to bacterial DNA via species-specific CpG motif recognition. Proceedings of the National Academy of Sciences.

[5] Haas T (2008). The DNA sugar backbone 2′ deoxyribose determines toll-like receptor 9 activation. Immunity.

[6] Di Pucchio T (2008). Direct proteasome-independent cross-presentation of viral antigen by plasmacytoid dendritic cells on major histocompatibility complex class I. Nature immunology.

[7] Fonteneau J-F (2003). Activation of influenza virus–specific CD4+ and CD8+ T cells: a new role for plasmacytoid dendritic cells in adaptive immunity. Blood.

[8] Jego G (2003). Plasmacytoid dendritic cells induce plasma cell differentiation through type I interferon and interleukin 6. Immunity.

[9] Poeck H (2004). Plasmacytoid dendritic cells, antigen, and CpG-C license human B cells for plasma cell differentiation and immunoglobulin production in the absence of T-cell help. Blood.

[10] Wu X, Gowda NM, Kumar S, Gowda DC (2010). Protein-DNA complex is the exclusive malaria parasite component that activates dendritic cells and triggers innate immune responses. J Immunol.

[11] Sharma S (2011). Innate immune recognition of an AT-rich stem-loop DNA motif in the Plasmodium falciparum genome. Immunity.

[12] Baccarella A, Fontana MF, Chen EC, Kim CC (2013). Toll-like receptor 7 mediates early innate immune responses to malaria. Infection and immunity.

[13] Liehl P (2014). Host-cell sensors for Plasmodium activate innate immunity against liver-stage infection. Nature medicine.

[14] Voisine C, Mastelic B, Sponaas A-M, Langhorne J (2010). Classical CD11c+ dendritic cells, not plasmacytoid dendritic cells, induce T cell responses to Plasmodium chabaudi malaria. International journal for parasitology.

[15] Spaulding E (2016). STING-Licensed Macrophages Prime Type I IFN Production by Plasmacytoid Dendritic Cells in the Bone Marrow during Severe Plasmodium yoelii Malaria. PLoS Pathog.

[16] DeWalick S (2007). Cutting edge: conventional dendritic cells are the critical APC required for the induction of experimental cerebral malaria. The Journal of Immunology.

[17] Yu X (2016). Cross-Regulation of Two Type I Interferon Signaling Pathways in Plasmacytoid Dendritic Cells Controls Anti-malaria Immunity and Host Mortality. Immunity.

[18] Pichyangkul S (2004). Malaria blood stage parasites activate human plasmacytoid dendritic cells and murine dendritic cells through a Toll-like receptor 9-dependent pathway. The Journal of Immunology.

[19] Pinzon-Charry A (2013). Apoptosis and dysfunction of blood dendritic cells in patients with falciparum and vivax malaria. The Journal of experimental medicine.

[20] Ibitokou S (2012). Peripheral blood cell signatures of Plasmodium falciparum infection during pregnancy. PloS one.

[21] Jangpatarapongsa K (2008). Plasmodium vivax parasites alter the balance of myeloid and plasmacytoid dendritic cells and the induction of regulatory T cells. Eur J Immunol.

[22] Kho S (2015). Preserved dendritic cell HLA-DR expression and reduced regulatory T cell activation in asymptomatic Plasmodium falciparum and P. vivax infection. Infection and immunity.

[23] Goncalves RM (2010). CD4+ CD25+ Foxp3+ regulatory T cells, dendritic cells, and circulating cytokines in uncomplicated malaria: do different parasite species elicit similar host responses?. Infect Immun.

[24] Urban BC (2006). Frequencies of peripheral blood myeloid cells in healthy Kenyan children with alpha+ thalassemia and the sickle cell trait. Am J Trop Med Hyg.

[25] Arama C (2011). Interethnic differences in antigen-presenting cell activation and TLR responses in Malian children during Plasmodium falciparum malaria. PLoS One.

[26] Montes de Oca M (2016). Type I Interferons Regulate Immune Responses in Humans with Blood-Stage Plasmodium falciparum Infection. Cell Reports.

[27] Woodberry T (2012). Low-Level Plasmodium falciparum Blood-Stage Infection Causes Dendritic Cell Apoptosis and Dysfunction in Healthy Volunteers. Journal of Infectious Diseases.

[28] Loughland JR (2016). Profoundly Reduced CD1c+ Myeloid Dendritic Cell HLA-DR and CD86 Expression and Increased Tumor Necrosis Factor Production in Experimental Human Blood-Stage Malaria Infection. Infection and immunity.

[29] Rockett, R. *et al*. A real-time, quantitative PCR method using hydrolysis probes for the monitoring of Plasmodium falciparum load in experimentally infected human volunteers. *Malaria journal* **10**, doi:10.1186/1475-2875-10-48 (2011). 10.1186/1475-2875-10-48 PMC3055851 21352599

[30] Barry S (1997). Roles of the N and C terminal domains of the interleukin-3 receptor α chain in receptor function. Blood.

[31] Colonna M, Trinchieri G, Liu YJ (2004). Plasmacytoid dendritic cells in immunity. Nature immunology.

[32] Grouard G (1997). The enigmatic plasmacytoid T cells develop into dendritic cells with interleukin (IL)-3 and CD40-ligand. Journal of Experimental Medicine.

[33] Dzionek A (2000). BDCA-2, BDCA-3, and BDCA-4: three markers for distinct subsets of dendritic cells in human peripheral blood. The Journal of Immunology.

[34] Cagnol S, Chambard JC (2010). ERK and cell death: Mechanisms of ERK‐induced cell death–apoptosis, autophagy and senescence. FEBS Journal.

[35] Ito T, Kanzler H, Duramad O, Cao W, Liu Y-J (2006). Specialization, kinetics, and repertoire of type 1 interferon responses by human plasmacytoid predendritic cells. Blood.

[36] Kobayashi KS, van den Elsen PJ (2012). NLRC5: a key regulator of MHC class I-dependent immune responses. Nature Reviews Immunology.

[37] Downs I, Vijayan S, Sidiq T, Kobayashi KS (2016). CITA/NLRC5: A critical transcriptional regulator of MHC class I gene expression. BioFactors.

[38] Tel J (2013). Human plasmacytoid dendritic cells efficiently cross-present exogenous Ags to CD8+ T cells despite lower Ag uptake than myeloid dendritic cell subsets. Blood.

[39] Wykes MN (2011). Rodent blood-stage Plasmodium survive in dendritic cells that infect naive mice. Proceedings of the National Academy of Sciences.

[40] Benkő, S., Kovács, E. G., Hezel, F. & Kufer, T. A. NLRC5 Functions beyond MHC I Regulation—What Do We Know So Far? *Frontiers in Immunology* **8**, doi:10.3389/fimmu.2017.00150 (2017). 10.3389/fimmu.2017.00150 PMC5313500 28261210

[41] Takayama S, Reed JC (2001). Molecular chaperone targeting and regulation by BAG family proteins. Nature Cell Biology.

[42] Rosati A (2009). BAG3 protein regulates caspase‐3 activation in HIV‐1‐infected human primary microglial cells. Journal of cellular physiology.

[43] Pombo DJ (2002). Immunity to malaria after administration of ultra-low doses of red cells infected with *Plasmodium falciparum*. The Lancet.

[44] Yang Y-C (1986). Human IL-3 (multi-CSF): identification by expression cloning of a novel hematopoietic growth factor related to murine IL-3. Cell.

[45] Kohara H (2007). Development of plasmacytoid dendritic cells in bone marrow stromal cell niches requires CXCL12-CXCR4 chemokine signaling. Blood.

[46] Yoneyama H (2004). Evidence for recruitment of plasmacytoid dendritic cell precursors to inflamed lymph nodes through high endothelial venules. International immunology.

[47] Piqueras B, Connolly J, Freitas H, Palucka AK, Banchereau J (2006). Upon viral exposure, myeloid and plasmacytoid dendritic cells produce 3 waves of distinct chemokines to recruit immune effectors. Blood.

[48] Zheng W (2009). CD4+ CD25+ Foxp3+ regulatory T cells prevent the development of Th1 immune response by inhibition of dendritic cell function during the early stage of Plasmodium yoelii infection in susceptible BALB/c mice. Folia parasitologica.

[49] Swiecki M, Wang Y, Gilfillan S, Colonna M (2013). Plasmacytoid dendritic cells contribute to systemic but not local antiviral responses to HSV infections. PLoS Pathog.

[50] Lehmann C (2014). Longitudinal analysis of distribution and function of plasmacytoid dendritic cells in peripheral blood and gut mucosa of HIV infected patients. Journal of Infectious Diseases.

[51] Haque A (2011). Type I interferons suppress CD4+ T‐cell‐dependent parasite control during blood‐stage Plasmodium infection. European journal of immunology.

[52] McCarthy JS (2011). A Pilot Randomised Trial of Induced Blood-Stage Plasmodium falciparum Infections in Healthy Volunteers for Testing Efficacy of New Antimalarial Drugs. PLoS ONE.

[53] Barber BE (2013). A prospective comparative study of knowlesi, falciparum, and vivax malaria in Sabah, Malaysia: high proportion with severe disease from Plasmodium knowlesi and Plasmodium vivax but no mortality with early referral and artesunate therapy. Clinical infectious diseases.

[54] Jerome K, Sloan D, Aubert M (2003). Measurement of CTL-induced cytotoxicity: the caspase 3 assay. Apoptosis.

[55] Field MA, Cho V, Andrews TD, Goodnow CC (2015). Reliably detecting clinically important variants requires both combined variant calls and optimized filtering strategies. PloS one.

[56] Dobin A (2013). STAR: ultrafast universal RNA-seq aligner. Bioinformatics.

[57] Li H (2009). The sequence alignment/map format and SAMtools. Bioinformatics.

[58] Anders, S., Pyl, P. T. & Huber, W. HTSeq–a Python framework to work with high-throughput sequencing data. *Bioinformatics*, Anders S, Pyl PT, Huber W. HTSeq—a Python framework to work with high-throughput sequencing data. *Bioinformatics* **31**, 166–169, doi:10.1093/bioinformatics/btu638 (2015). 10.1093/bioinformatics/btu638 PMC4287950 25260700

[59] Love MI, Huber W, Anders S (2014). Moderated estimation of fold change and dispersion for RNA-seq data with DESeq2. Genome biology.

