# Supplementary material for: Plasmacytoid dendritic cells appear inactive during sub-microscopic Plasmodium falciparum blood-stage infection, yet retain their ability to respond to TLR stimulation
Source: Sci Rep. 2017 Jun 1;7:2596. doi: 10.1038/s41598-017-02096-2 (PMC5453946; doi:10.1038/s41598-017-02096-2)
Supplement: Supplementary file 1 — Supplementary Information [file 41598_2017_2096_MOESM1_ESM.pdf]

Plasmacytoid dendritic cells appear inactive during sub-microscopic *Plasmodium falciparum* blood-stage infection, yet retain their ability to respond to TLR stimulation.

Jessica R. Loughland[1*], Gabriela Minigo[1], Derek S. Sarovich[1,2], Matt Field[3], Peta E. Tipping[1€], Marcela Montes de Oca[4], Kim A. Piera[1], Fiona H. Amante[4], Bridget E. Barber[1,6], Matthew J. Grigg[1,6], Timothy William[6,7], Michael F. Good[8], Denise L. Doolan[3,4], Christian R. Engwerda[4], Nicholas M. Anstey[1,5], James S. McCarthy[4+], Tonia Woodberry[1+].

[1]Menzies School of Health Research, Darwin, Australia and Charles Darwin University, Darwin, Australia; [2]Centre for Animal Health Innovation, Faculty of Science, Health, Education and Engineering, University of the Sunshine Coast, Sippy Downs, Queensland, Australia; [3]Australian Institute of Tropical Health and Medicine, James Cook University, Cairns, Australia; [4]QIMR Berghofer Medical Research Institute, Brisbane, Australia; [5]Royal Darwin Hospital, Darwin, Australia; [6]Infectious Diseases Unit, Queen Elizabeth Hospital, Kota Kinabalu, Sabah, Malaysia; [7]Sabah Department of Health, Kota Kinabalu, Sabah, Malaysia; [8]Griffith University, Gold Coast, Australia.

* jessica.loughland@menzies.edu.au

+ These authors contributed equally to this work.

€ Present Address: Royal Darwin Hospital, Darwin, Australia.

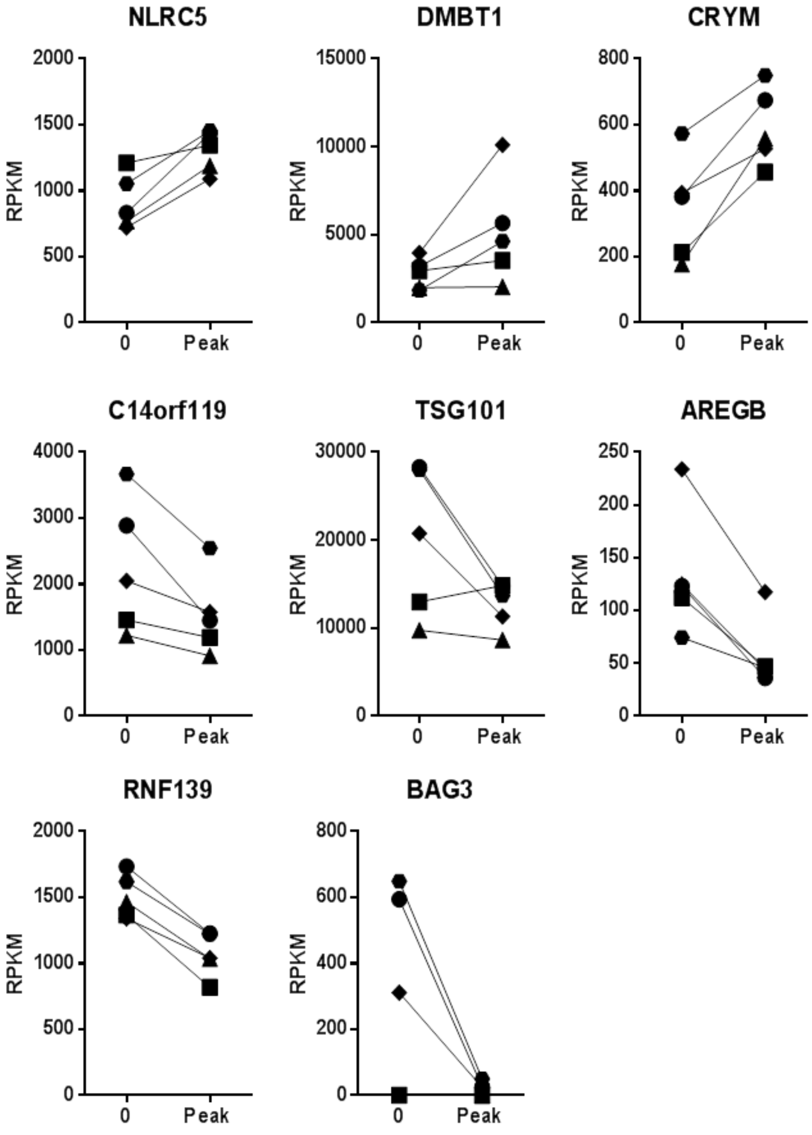

Supplementary Figure 1 Summary of differential gene expression. Longitudinal pDC gene expression changes in five participants, between day 0 and peak parasitemia (day 7-8). Represented genes had an FDR<0.07, each symbol represents one participant.
